# RNAseq of Osteoarthritic Synovial Tissues: Systematic Literary Review

**DOI:** 10.3389/fragi.2022.836791

**Published:** 2022-05-25

**Authors:** Logan Moore, Zui Pan, Marco Brotto

**Affiliations:** Bone Muscle Research Center, College of Nursing and Health, University of Texas at Arlington, Arlington, TX, United States

**Keywords:** osteoarthritis, joint, synovial, RNAseq, biotechnology

## Abstract

Osteoarthritis (OA) is one of the most common causes of disability in aged people, and it is defined as a degenerative arthropathy, characterized by the disruption in joint tissue. The synovium plays a vital role in maintaining the health of the joint by supplying the nutrients to the surrounding tissues and the lubrication for joint movement. While it is well known that all the joint tissues are communicating and working together to provide a functioning joint, most studies on OA have been focused on bone and cartilage but much less about synovium have been reported. The purpose of this review was to investigate the current literature focused on RNA sequencing (RNAseq) of osteoarthritic synovial tissues to further understand the dynamic transcriptome changes occurring in this pivotal joint tissue. A total of 3 electronic databases (PubMed, CINHAL Complete, and Academic Complete) were systematically searched following PRISMA guidelines. The following criteria was used for inclusion: English language, free full text, between the period 2011–2022, size of sample (*n* > 10), study design being either retrospective or prospective, and RNAseq data of synovial tissue from OA subjects. From the initial search, 174 articles, 5 met all of our criteria and were selected for this review. The RNAseq analysis revealed several differentially expressed genes (DEGs) in synovial tissue. These genes are related to the inflammatory pathway and regulation of the extracellular matrix. The MMP family, particularly MMP13 was identified by three of the studies, indicating its important role in OA. IL6, a key contributor in the inflammation pathway, was also identified in 3 studies. There was a total of 8 DEGs, MMP13, MMP1, MMP2, APOD, IL6, TNFAIP6, FCER1G, and IGF1 that overlapped in 4 out of the 5 studies. One study focused on microbial RNA in the synovial tissue found that the microbes were differentially expressed in OA subjects too. These differentially expressed microbes have also been linked to the inflammatory pathway. Further investigation with more clinical gene profiling in synovial tissue of OA subjects is required to reveal the causation and progression, as well as aid in the development of new treatments.

## Introduction

Osteoarthritis (OA) is a chronic, debilitating joint disease. It is clinically defined as disruption and potential loss of joint cartilage, as well as changes to other joint tissues (Neogi and Zhang 2013; Johnson and Hunter 2014; Glyn-Jones et al., 2015; Vina et al., 2018). OA can occur in any population, and the causation is multifactorial such as age, sex, obesity, previous history of injury, and genetic disposition (Blagojevic et al., 2010; Johnson and Hunter 2014; Glyn-Jones et al., 2015). As a painful disease, OA is one of the most common causes of disability, especially in aged people (Neogi and Zhang 2013; Johnson and Hunter 2014; Glyn-Jones et al., 2015). Currently, there is no known cure and limited treatments for OA patients, including lifestyle changes, exercise, pain medication, and in the most severe cases surgery Click or tap here to enter text (Johnson and Hunter 2014; Bannuru et al., 2019). The prevalence of this disease is predicted rising due to the rise of obesity rates and the aging population (Blagojevic et al., 2010; Johnson and Hunter 2014). The CDC estimates by the year of 2040 78 million US adults will be affected by OA, an increase by 20 million as compared to now (Barbour et al., 2017). This places a major public health burden with annual indirect cost ranging from approximately 1,400 to 22,000 USD per patient (Johnson and Hunter 2014; Xia et al., 2015).

It is well established that all the joint tissues are communicating and working together to provide a functioning joint. Hyaline cartilage is the dense connective tissue that forms a smooth articulating surface for joints (Rovenský and Payer 2009; Goldring 2020). The hyaline cartilage is avascularized and is dependent on the nutrients provided by the synovium (Rovenský and Payer 2009; Smith and Wechalekar 2015). The synovium is a soft tissue that lines the joint cavity except for the cartilage (Smith and Wechalekar 2015; Veale et al., 2017). It contains a rich network of blood and lymphatic vessels in which provide the nutrients for homeostasis in the joint (Veale et al., 2017). It also secretes synovial fluid, comprised of hyaluronan, lubricin, lactoferrin and proinflammatory and anti-inflammatory cytokines (Guillen et al., 1998; Petty and Cassidy 2005; Smith and Wechalekar 2015; Veale et al., 2017; Stark et al., 2019). The synovial fluid is a highly viscous material that aids in the lubrication of the joint space for smooth articulation (Petty and Cassidy 2005; Stark et al., 2019). Thus, the synovial tissues are vitally important in protection, maintenance, and homeostasis of the joint. While hyaline cartilage is typically at the forefront of OA research as it is seen as the most effected joint tissue, yet, much less about synovium have been reported.

Comparing the gene expression pattern in synovial cells from OA patients to that from healthy subjects can provide important information to understand the cellular mechanism underlying the pathology of OA, which may shed light to identification of new therapeutic targets. The complete set of gene expression pattern, i.e., transcriptome (Transcriptome, 2014), can be obtained by high-throughput techniques such as microarrays and RNA sequencing (RNAseq) (O’Leary et al., 2016). RNAseq was first introduced over a decade ago and is still considered the next generation of transcriptome analysis (Wanget al., 2009; Kukurba and Montgomery 2015). Due to the development of faster and cheaper sequencing technique, RNAseq becomes to be a standard research tool to provide whole transcriptome-wide analysis (Kukurba and Montgomery 2015; Wang et al., 2009; Stark et al., 2019). It allows researchers to investigate the regulation of genes by their level of expression, also if there are changes or mutations in a certain transcriptome (Kukurba and Montgomery 2015).

This review is to collect and analyze the current literature on the dynamic transcriptional changes occurring in osteoarthritic synovial tissue, which may assist to identify the potential causation of OA and to provide targets for therapies.

## Methods

Three electrotonic online databases, PubMed, CINHAL Complete, and Academic Search Complete, were systematically searched using the guidelines set forth by the Preferred Reporting Items for Systematic Reviews and Meta-analysis (PRISMA) (Moher et al., 2015). The initial, investigatory search was completed at the conception of this study, February 2021. The final systematic search was conducted in May 2022. The following key words were used for the search string: “Osteoarthritis,” “RNA sequencing,” “RNAseq,” “RNA-seq,” “Synovial,” Synovium”. The key words were joined by either “AND” or “OR”.

For inclusion in this review articles needed to meet the following criteria: 1) English Language, 2) Free full text available, 3) Study design followed either retrospective or prospective design, 4) between the period 2011 and 2022, 5) sample population groups greater than 10 samples, 6) RNAseq performed on osteoarthritic synovial tissue.

Articles were excluded if they did not meet the stated inclusion criteria. A priori to the search it was deemed the articles needed to be of the utmost caliber and rigor, therefore non-peer-reviewed articles, newspapers, and editorials were excluded. Only articles of the English language were included, to ensure proper interpretation from the reviewer. Since the review is a systematic review, all systematic reviews, narrative reviews, or meta-analysis were excluded. All abstracts, study protocols, and pilot data were excluded. A prior it was determined only completed articles with groups larger than 10 participants per group were included, to ensure precision of results.

Following each search, the results were downloaded for importation into a reference manager. The results were downloaded in two formats.bib and.csv. The reference manager was used for duplicate removal and storage of full text. To ensure the reference manager was correct, all resulting articles were also managed in a separate spreadsheet. After duplicate removal, a broad scan of titles and abstracts was conducted eliminating any articles that were obviously not meeting the inclusion/exclusion criteria. The resulting full text of the articles was reviewed for inclusion.

The final studies were assessed for bias. The resulting bias assessment did not dictate inclusion or exclusion in this review, but rather serves as further information on the quality of the articles included. Either the Revised Cochrane risk-of-bias tool for randomized trials (RoB2) or The Risk of Bias in Non-randomized Studies of Interventions (ROBINS-I) were used to determine bias based on their classification type of clinical study either being retrospective, clinical case series, prospective or randomized control trials as indicated by The National Health and Medical Research Council (NHMRC. 2000) (Sterne et al., 2019; 2016). RoB2 was used for randomized control studies and ROBINS-I was used for non-randomized control studies. The tool robvis was used for the visualization of the results (McGuinness and Higgins 2021).

## Results

**FIGURE 1:**
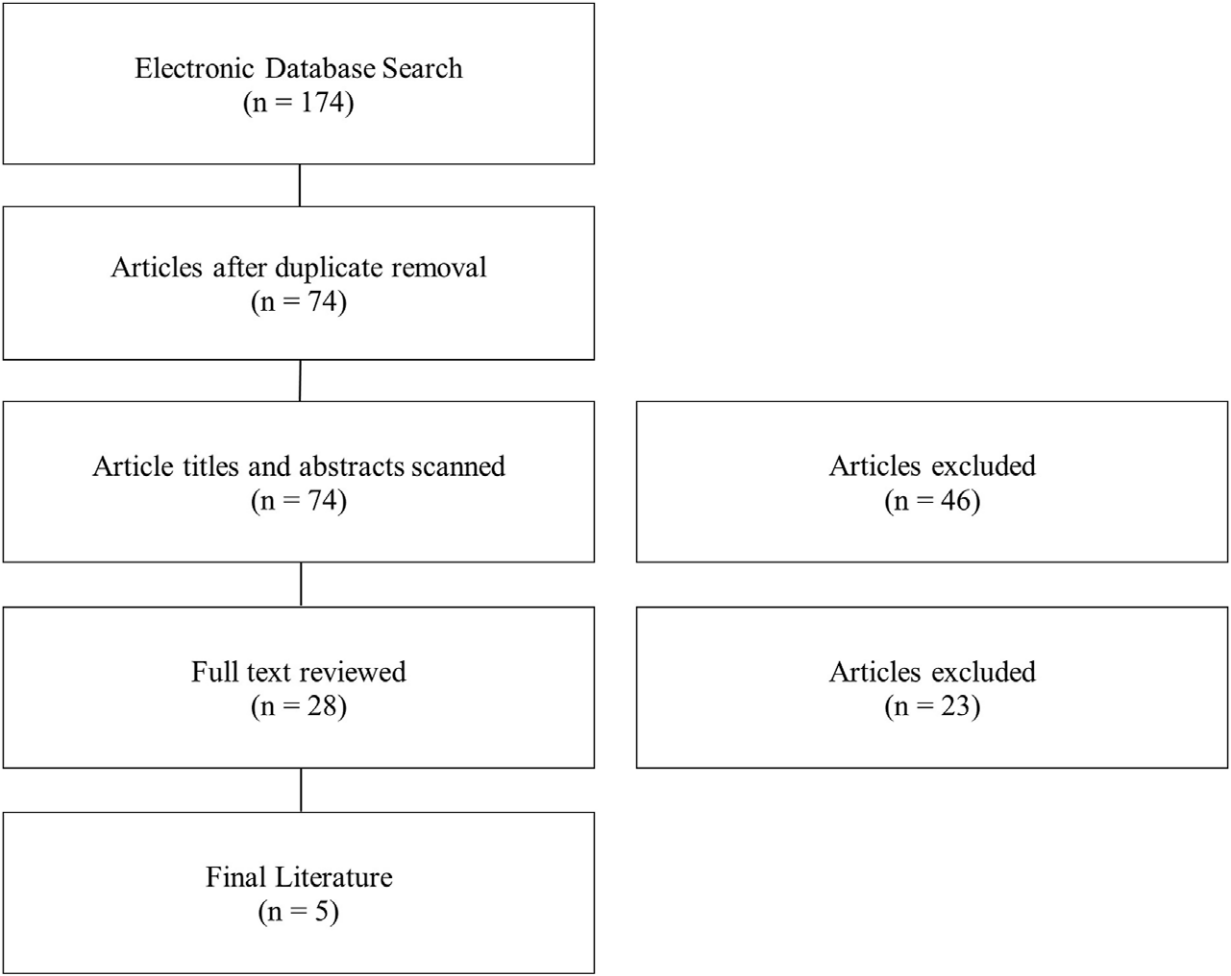
Flow diagram of literary search strategy and eligibility.

Through the initial search, a total of 174 articles were found (Figure 1). Duplicate removal removed 100 articles. This shows that there was consistent overlap in the literature between search strings and without variation of the search string articles may have been missed. The titles and abstracts were broadly reviewed. A total of 46 articles were removed for not meeting inclusion/exclusion criteria. The remaining articles (*n* = 28) had the full text reviewed. After full text review, 5 articles met the inclusion/exclusion criteria set a priori. Exclusion from this review mainly resulted from RNAseq not being performed on osteoarthritic synovial tissue, incomplete data, or not enough samples per group. The search was updated periodically throughout the drafting of this manuscript and the final literature search was in 02.

The 5 studies included in this review were all published between 2020 and 2022 (Table 1). Three articles were published from the United States (United States) (Tsai et al., 2020; Li and Zheng 2020; McCoy et al., 2020), one article was jointly published from Germany and the United Kingdom (Steinberg et al., 2021), and one article jointly published from China and the United States (Huang et al., 2022). The articles were either defined as a retrospective or prospective. The two retrospective studies obtained their RNAseq data from the NCBI GEO DataSets (https://www.ncbi.nlm.nih.gov/gds), in which one used the data from United States Human OA subjects (Li and Zheng 2020) and the other used Australian Human OA subjects (Tsai et al., 2020). In the three prospective studies, one used a post-traumatic equine model, and two used synovial tissue obtained prior to arthroplasty of either the hip or knee in human subjects (Steinberg et al., 2021; Huang et al., 2022).

**TABLE 1:** Data summary of the 5 selected articles for review.

Authors	Year	Country	Type of Study	Sample composition
Tsai et al.	2020	United States	Retrospective	14 Human OA
Li et al.	2020	United States	Retrospective	20 Human Knee OA
McCoy et al.	2020	United States	Prospective	11 Post-traumatic OA equine
Steinberg et al.	2021	Germany, United Kingdom	Prospective	90 Human knee OA
Huang et al.	2021	China, United States	Prospective	14 Human Knee OA

When the synovium from the OA patients (*n* = 20) were compared to that from the healthy controls, 372 genes were identified as differential expression genes (DEG) (Li and Zheng 2020). Of those genes 188 had increased expression and 184 had decreased levels of expression (Li and Zheng 2020). Using the UniProt system, the authors identified 10 DEGs related to the inflammatory pathway that were significantly altered (Li and Zheng 2020). These DEG are: Apolipoprotein D (APOD), Complement CLq subcomponent subunit B (C1QB), N-formyl peptide receptor 3 (FPR3), Histone cluster 1 H3 family member b (HIST1H3B), Interferon epsilon (IFNE), Macrophage scavenger receptor typee I (MSR1) , Lymphokine-activated killer T-cell-originated protein kinase (PBK), Tumor necrosis factor-inducible gene 6 protein (TNFAIP6), Triggering receptor expressed on myeloid cells I (TREM1), and V-set and immunoglobulin domain-congaing protein 4 (VSIG4). Li et al. also found there are 7 genes that were significantly altered that contribute to the functionality of the Extracellular Matrix (ECM). Those 7 genes are Myocilin (MYOC), Amelotin (AMTN), Chitinase-3-like protein 2 (CHI3L2), Prolyl endopeptidase Fibroblast activation protein alpha (FAP), Leucine-rich repat-containing protein 15 (LRRC15), Matrix Metallopeptidase-13 (MMP13), TNFAIP6 (C. Li and Zheng 2020).

In single cell RNAseq conducted by Huang et al. they profiled over 93,000 synovial cells from 14 knee OA individuals (female, age: 70.7 ± 5.9 years; BMI: 25.9 ± 4.4 kg/m^2^). They were able to identify 7 distinct cell types: Fibroblasts (59%), antigen presenting cells (APCs) (13.6%), T cells (11.4%), endothelial cells (ECs) (10%), mural cells (3%), B cells (1.8%) and mast cells (1%) along with 43 DEGS. The APCs had 4 cell subtypes: Transitional macrophages, fibrotic immune regulated macrophages, interferon stimulated macrophages, and S100A8/9^hi^ macrophages. These macrophage cell types showed high expression in genes related to inflammation and the inflammatory pathway, such as IL6 (Interterluekin-6), CCL3 (C-C Motif Chemokine Ligand 3), CCL3L1 (C-C Motif Chemokine Ligand 3 Like 1), IL1A (Interleukin-1 alpha) IL1B (Interleukin-1 Beta), TLR2 (Toll-like receptor 2)*.* However, transitional macrophages also showed high expression in inflammation resolving genes IGF1 (Insulin Like Growth Factor 1) and MRC1 (Mannose receptor C-type 1).

McCoy et al. used a post-traumatic OA equine model (*n* = 11) to demonstrate the effects of OA on the synovium (McCoy et al., 2020). In this study, a larger amount of DEGs was reported as compared to Li et al. It was found that there were 397 genes that had been upregulated, and 365 genes down regulated (McCoy et al., 2020). Using a Markov clustering algorithm 213 DEGs were able to be assigned to 28 unique clusters. Nine of the clusters had equal to or greater than 10 DEGs, detailed in the Supplementary Table S1. There were significant alterations in gene expression pathways for ECM organization and protein metabolism (McCoy et al., 2020).

Based upon RNAseq data from the synovial lining of OA patients (*n* = 90), Steinberg et al. identified two groups of OA patients, low-grade and high-grade OA (Steinberg et al., 2021). Steinberg et al. defined low-grade OA as having largely intact cartilage as compared to the degraded tissue in the high-grade group. The authors showed that there are DEGs in the inflammatory pathway between high-grade OA and low-grade OA. Furthermore, the low-grade OA group can be subdivided into two subgroups based on the changes in transcription in the ECM pathway. The authors developed a predictive tool to detect low-grade OA in the knee based on 7 DEGs [MMP1, MMP2, MMP13, APOD, IL6, CYTL1 (Cytokine-like 1), C15orf48 (Chromosome 15 open reading frame 48)] related to the inflammatory pathway (Steinberg et al., 2021).

Using GEO RNAseq data, Tsai et al. found 299 bacterial species in OA synovial biopsies (*n* = 14) (Tsai et al., 2020). A total of 84 bacterial species were found in healthy synovial biopsies, however not all the microbial species were found in the synovium of OA samples. There were 43 microbes to be found differentially abundant in the OA samples as compared to the healthy, detailed in the Supplementary Table S1. Of the 43 differentially abundant microbes, 27 of them were species of *Pseudomonas*. The bacterium found in OA synovial biopsies can be linked to immune signatures and immune cell types (Tsai et al., 2020).

**FIGURE 2:**
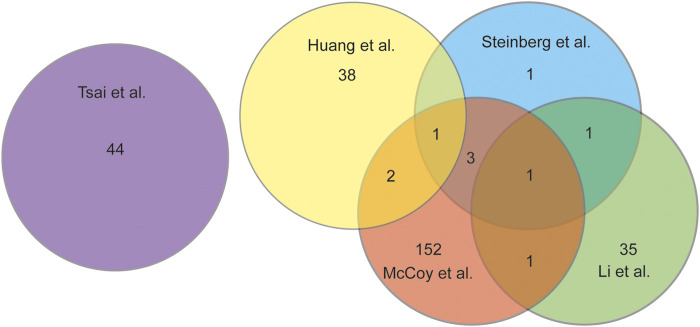
Venn diagram analysing the overlap of DEGs discussed in each study chosen for this review.

Comparing the 5 studies selected there were only 8 DEGs that were represented in multiple studies, with 2 DEGs being represented in 3 of the 5 studies (Figure 2). Tsai et al. had no overlapping DEGs as this study analyzed microbial RNA as compared to the others. The 8 DEGs that were represented in at least 2 studies were: MMP1, MMP2, MMP13, APOD, TNFAIP6, IL6, FCER1G, and IGF1. Three of the overlapping DEGs, APOD, TNFAIP6, and IL6 are all genes that have been found to regulate the pro-inflammatory pathway (Li and Zheng 2020; McCoy et al., 2020; Steinberg et al., 2021; Huang et al., 2022). IL6 was differently expressed in 3 of the 5 studies and is a key contributor to modulating inflammation in both acute and chronic inflammation (McCoy et al., 2020; Steinberg et al., 2021; Huang et al., 2022; Safran et al., 2022). IL6 has a crucial role in the pathology of OA, as it induces MMP13 and through pathway signaling reduces the sensitization of IGF1 (Wiegertjes et al., 2020). IGF1 was shown to be highly expressed having a role in the inflammation resolving pathway, as well as having a role in enhanced extracellular matrix production and inhibition of apoptosis in chondrocytes. (McCoy et al., 2020; Wen et al., 2021; Huang et al., 2022). The gene MMP13 was represented in 3 of the 5 studies included. MMP13 is a gene that encodes a peptidase in the matrix metalloproteinases (MMP) family (Safran et al., 2022). MMP13 when synthesized must be proteolytically processed to become a matured protease (Salerno et al., 2020). This activated protease is known to cleave type II collagen (Garnero 2007; O’Leary et al., 2016; Salerno et al., 2020). The MMP family of genes has been shown to be upregulated in OA patients and linked to the cartilage destruction through the breakdown of collagen fibers (Davidson et al., 2006; McCoy et al., 2020). In OA samples the MMP family was seen to be upregulated by 8.5 to 12.7fold (McCoy et al., 2020).

**FIGURE 3:**
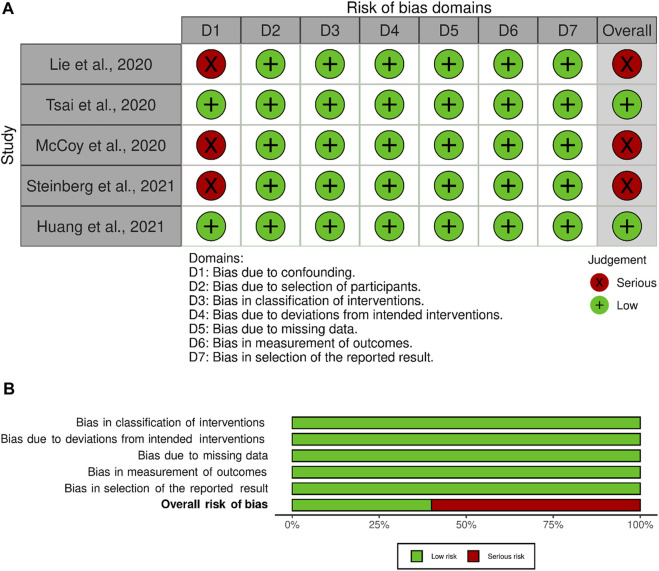
Bias Reporting, **(A)** Bias domains for ROBINS-I for the included studies. **(B)** Total score for each ROBINS-I score.

Risk of bias was assessed for each included article. All remaining articles were determined to be non-randomized control trials. Therefore, only the ROBIN-1 assessment tool was used. A priori to assessing the articles it was determined that the confounding domains were gender, age, previous history of injury, and intervention pre-sampling of the synovial tissue. Of the 5 articles selected for this review two articles scored a low risk of bias (Figure 3) (Tsai et al., 2020; Huang et al., 2022). The remaining 3 articles were found to have a serious risk of bias due to confounding domains (Steinberg and Brooks. 2018; Li and Zheng 2020; McCoy et al., 2020). To score a serious risk of bias in domain 1, the articles either did not account or report gender differences, previous history of injury, or intervention pre-sampling.

## Discussion

It is evident that the RNAseq is a relative new technique in the field of OA research, as all the articles selected for this study are between 2020 and 2021, and only 4 articles met the inclusion/exclusion criteria. In the 4 articles, each showed significant alterations in gene expression in the synovial tissue of OA subjects. The genes shown to be differentially expressed have contributing roles in the destruction of collagen fibers and inflammation of the joint. The understanding of the mechanisms and pathways which contribute to the progression of OA will aid in the development of new therapies, interventions, and detection methods.

Synovial tissue plays a pivotal role in the causality and progression of OA. This is illustrated by the dynamic changes found in the transcriptome produced through RNAseq. Each study selected for this review showed significant DEGs as compared to the healthy controls. In a PTOA equine model it showed that there were as many as 762 changes in the transcriptome, and in a human OA model there were as many as 372 DEGs. There was 8 DEGs that were discussed in 2 or more of the studies included, and 2 DEGs that was discussed in 3 out of the 5 studies. The 8 DEGs (MMP1, MMP2, MMP13, FAPOD, IL6, TNFAIP6, FCER1G, IGF1) require future investigation as potential biomarkers in the onset or progression of OA (Table 2).

**TABLE 2:** The 8 DEGs found to overlap in the 5 studies (Safran et al., 2022).

Gene		Article found	Description
MMP13	Matrix Metallopeptidase 13	Li et al., McCoy et al., Steinberg et al.	Associated with the breakdown of the extracellular matrix. Member of the M10 family of matrix metalloproteinases. The associated mature protease cleaves type II collagen and is involved in the turnover of articular cartilage
MMP1	Matrix Metallopeptidase 1	McCoy et al., Steinberg et al.	Associated with the breakdown of the extracellular matrix. Member of the M10 family of matrix metalloproteinases. The associated mature protease breakdown interstitial collagens (type I, II, and II)
MMP2	Matrix Metallopeptidase 2	McCoy et al., Steinberg et al.	Associated with the breakdown of the extracellular matrix. The associated protease differs from other MMP family members as it is activated on the cell membrane either extracellularly or intracellularly. It is responsible for the breakdown of collagen type IV and V and elastin
APOD	Apolipoprotein D	Li et al., Steinberg et al.	Encodes for a high-density lipoprotein, which is a family member of the lipocalins. The associated lipoprotein is involved in the binding and transport of bilin
IL6	Interleukin 6	McCoy et al., Steinberg et al., Huang et al.	Encodes for a pro-inflammatory cytokine, and the protein is produced at sites of acute and chronic inflammation. It is associated with inducing the acute phase immune response
TNFAIP6	Tumor Necrosis Factor Alpha-Inducible Protein 6	Li et al., McCoy et al.	Associated with the maintenance of the extracellular matrix and can be induced through pro-inflammatory cytokines. The associated secretory protein is a member of the hyaluronan-binding protein family
FCER1G	Fc Epsilon Receptor IG	McCoy et al., Huang et al.	Encodes for an adaptor protein which produces activation signaling in immunoreceptors. Contributes to the cell differentiation of T-cells
IGF1	Insulin Like Growth Factor 1	McCoy et al., Huang et al.	Associated with mediating growth and development. The associated protein activates tyrosine kinase activity, and downstream activates the P13 K-AKT/PKB and the Ras-MAPK pathways

The regulation of the MMP family and more specifically MMP13 has already become a topic of interest as seen in recent studies (Salerno et al., 2020; Bai et al., 2020; Wang et al., 2013; Li et al., 2017; Murphy and Lee 2005). Cells found in the synovium and in cartilage both are found to express MMP13 (Murphy and Lee 2005; Davidson et al., 2006). In the Biological Process (GO) found in the STRING network, the terms related to MMP13 are extracellular matrix organization, extracellular matrix disassembly, collagen catabolic process, skeletal system development, and multicellular organismal process (MMP13 Protein, 2021). Davidson et al. confirms the results of these studies by showing a significant upregulation in both synovium and cartilage using quantitative PCR (Davidson et al., 2006). MMP13 plays a central role in the degradation of extracellular matrix by degrading collagen type I, II, III, IV, XIV, and X, with its highest activity being collagen type II (Murphy and Lee 2005; MMP13 Gene, 2021). MMP13 is thought to be an important factor in the early onset and progression of OA (Davidson et al., 2006; Wang et al., 2013; Sato et al., 2006). MMP13 is now a targeted gene in potential therapies (Salerno et al., 2020; Wang et al., 2013). In a study conducted by Wang et al., MMP13 is shown be a significant regulator in OA progression (M. Wang et al., 2013). Using a MMP13 knock out mouse, after OA induction by meniscal-ligamentous injury the knockout (Mmp13Col2ER) mice showed significant decrease in degeneration of cartilage/OA progression at 8, 12, and 16 weeks (Wang et al., 2013). The potential of being able decrease or eliminate the destruction of cartilage through regulating MMP13 and the MMP family, could provide clinical and preventative treatments of OA. This implication would have a major impact on the future of OA research and most importantly the wellbeing of the effected patients. While in cartilage the down regulating the production of the MMP family and MMP13 may have beneficial effects to the development of OA it may have detrimental effects in other joint tissues. It was shown recently that MMP13 plays a vital role in osteogenic differentiation and by knocking out MMP13 osteogenic differentiation decreased (Arai et al., 2021). This presents problems in global inhibition of MMP13, and the need to create tissue specific genetic inhibitors.

Inflammation of the synovium or synovitis is a common pathology in OA patients (Ayral et al., 2005; Tsuchida et al., 2014; Wiegertjes et al., 2020). Of the 8 overlapping DEGs found, 3 DEGs (APOD, TNFAIP6, and IL6) had roles in the pro-inflammatory pathway. IL6 is a common cytokine found in the pathogenesis of various inflammatory diseases, and recently has been a target in the treatment of OA (Laavola et al., 2018; Wiegertjes et al., 2020). In addition to the pro-inflammatory effects, IL6 has been shown to contribute to the disruption in the extracellular matrix in the cartilage of OA subjects. IL6 induces A Disintegrin and Metalloproteinase (ADAMTS) and the MMP family including MMP13, which degrades the extracellular matrix and collagen in the cartilage (Laavola et al., 2018; Wiegertjes et al., 2020). In a study conducted by Laavola et al., they examined the effect of inhibition of IL6 through the treatment with stilbenoids. It was shown that stilbenoids inhibits the expression of IL6 and reduce the levels of MMPs (Laavola et al., 2018). Increased expression of IL6 can also lead to IGF-1 desensitization (Wiegertjes et al., 2020). IGF-1 is critical to the repair of the cartilage, as IGF-1 inhibits the cartilage catabolism and promotes cartilage regeneration. (Wen et al., 2021). More research is needed, however the inhibition of IL6 has potential in being a superb mechanism in the treatment of OA, through the decreasing MMP and potentially restoring the sensitivity of IGF-1.

The linkage between the gut microbiome and gut permeability leading to musculoskeletal disease has been widely researched. It has been shown that gut permeability is increased in obese and aging populations, which are both predisposed to OA (Nagpal et al., 2018; Portincasa et al., 2021). There is a possible linkage between pathogenic bacteria leaking from the gut migrates into the synovium to create systemic inflammation leading to the onset of OA (Collins et al., 2015; Ulici et al., 2018; Tsai et al., 2020). The 8 overlapping DEGs that were found have a pivotal role in modulating inflammation and degradation of cartilage, and more so the inflammatory pathway was discussed to be differently expressed in all 5 articles chosen for this review. Interestingly, the differentially abundant bacteria found by Tsai et al. were majority from the species *Pseudomonas* (Tsai et al., 2020). There were also *E. Coli* differentially expressed. Both are gram-negative bacterium, which produces lipopolysaccharide (LPS). LPS has been identified as an important factor in the development of OA, for the proinflammatory response (Huang and Kraus 2016). It was shown recently by Mendez et al. that in a post-traumatic OA mouse model given the mouse an injection of LPS prior to injury increased the development of OA (Mendez et al., 2020). The hypothesis that bacteria can increase the risk and development of OA can be shown by Ulici et al., who showed that in germ-free mice the development of OA was reduced (Ulici et al., 2018). Tsai et al. showed that 299 bacterial species were in the synovial biopsies. Being able to limit the leakage of bacteria from the gut and the type of species could modulate the inflammation and progression of OA. Further research is required to illustrate the relationship between bacteria, bacterial components, and the development of OA.

OA is a chronic joint arthropathy disrupting multiple joint tissues, and the disruption in the transcriptome in the synovium plays a significant role in the onset and progression of this disease. As discussed earlier synovial tissue provides cartilage with nutrients, as well as regulates the synovial fluid. Through understanding the many dynamic changes in the transcriptome of the synovial tissue, researchers will have a greater level of discernment on the mechanisms of OA. The 5 articles that were selected in this review showed transcriptional changes in the inflammatory pathway as well as the extracellular matrix pathway. These two pathways have been a common focus in OA research, and still more research into how modulating these pathways can decrease and prevent the detrital, and painful effects of OA is needed.

There were 2 studies selected for this review that passed all seven domains in the bias assessment. The three other articles had a high risk of bias due to confounding variables. In future research, confounding variables need to be considered, as the causation of OA is multifactorial. Accounting for these variables, such as age, gender, ethnicity, or history of injury could provide insights into new genetic links and the identification of new biomarkers for a certain subset of OA patients. Each of these studies had the samples taken at a singular time point, further research needs to be developed in longitudinal studies to show the changes in transcriptional factors over the progression of the disease.

Transcriptional analysis is important in understanding disease mechanisms, as it gives information on the amplification of genes. The regulation of these genes effects the quality, composition, and number of certain proteins in the body. Furthermore, it is important to correlate the transcriptional profile with proteomic, lipidomic, and metabolic profiles to encompass the entire mechanistic effect of the disease. In a proteomic analysis of human OA synovial fluid 677 proteins were identified. Forty percent of the proteins were extracellular, 12% had a molecular function of extracellular matrix structural constituent, and 13% of the biological processes were involved in the immune response (Balakrishnan et al., 2014) In a recent lipidomic profile and metabolomic analysis of OA synovial membrane it was found that there were 53 significantly modulated lipids (Rocha et al., 2021). Glycerophospholipids (GP) were shown to be significantly elevated and have been correlated in articular inflammation and joint degeneration (Brouwers et al., 2015; Rocha et al., 2021). Rocha et al. also showed an increase in metabolomics associated with oleic acid, arachidonic acid, and lysophosphatidic acid. Arachidonic acid has been linked to the recruitment and activation of immune cells in the early phase of inflammation (Lawrence et al., 2002; Rocha et al., 2021). These results are congruent with the findings in the 5 selected articles in which there were a significant change in genes responsible for regulating the extracellular matrix and inflammatory pathway.

It has already been shown that the prevalence of OA is on the rise; the COVID-19 pandemic situation could increase the OA prevalence to an even higher levels (Blagojevic et al., 2010; Johnson and Hunter 2014). Sedentary behavior and the level of sedentariness has been directly linked to the onset and severity of OA symptoms (Daste et al., 2021). Through the global shut down physical activity levels have decreased, and sedentary behavior increased (Stockwell et al., 2021). This decrease in physical activity will have adverse effects in years to come placing a greater public health need, therefore continued investments in research efforts into understanding the mechanisms and pathways of OA is urgent.

## Conclusion

OA is a multi-faceted disease in which causation and progression depends not only on a single joint tissue, but all joint tissues and body as a whole. Through the understanding that there are significant transcriptional changes occurring in the synovium and synovial tissues, further research can be developed to identify potential biomarkers for detection and for treatments. There were 8 DEGS (MMP13, MMP1, MMP2, APOD, IL6, TNFAIP6, FCER1G, IGF1) that overlapped in 3 out of the 5 articles selected for this review. These 8 genes offer great potential in being recognized as biomarkers in OA, as these genes play a significant role in the regulation of the extracellular matrix and the inflammatory pathway. The creation of longitudinal studies that have a direct focus on how these genes are differently expressed will aid in creation of early detection screens and targets in gene therapy. The role in which the microbiome plays by inspiring inflammation and causing alterations in the synovium and joint tissues needs to be further explored. Through understanding the dynamic changes in the differentially expressed bacteria the onset and progression of disease may be describe through environmental or dietary elements. The study of RNAseq of OA synovial tissue is relatively scarce but is desperately needed. More RNAseq datasets of synovial tissues during the progression of OA and among different subsets of OA patients will provide molecular mechanisms and identify genetic links to the causation and progression of OA.

## Data Availability

The original contributions presented in the study are included in the article/Supplementary Material, further inquiries can be directed to the corresponding author.
